# Trigonometry in daily ultrasound practice

**DOI:** 10.1186/s13054-018-2282-8

**Published:** 2018-12-22

**Authors:** Alessandro De Cassai, Ludovica Sandei, Michele Carron

**Affiliations:** Department of Medicine - DIMED, Section of Anesthesiology and Intensive Care, University of Padova, Via V. Gallucci, 13, 35121 Padova, Italy

We have read with interest the editorial by Piton et al. [1] regarding application of Pythagoras’ theorem in central venous cannulation. Geometry is at the base of our daily life and we should apply his theorems and axioms to obtain the best result in our daily practice.

**Fig. 1:**
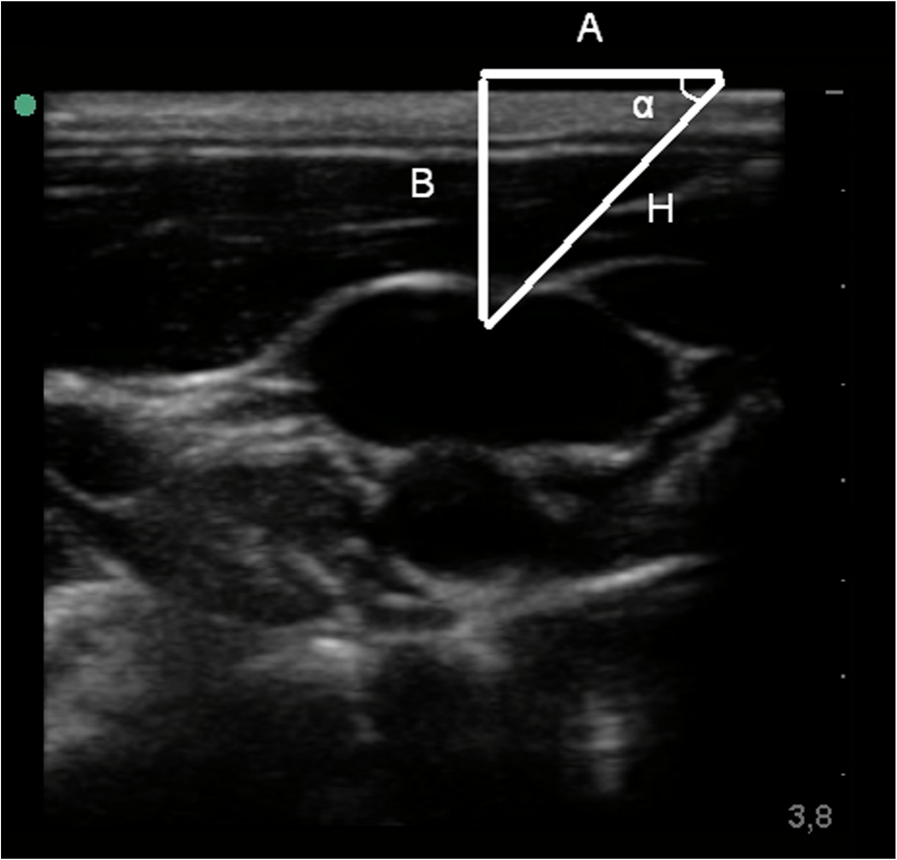
Internal jugular vein ultrasound anatomy. *H* hypotenuse; A and B are catheti of the triangle

We believe that trigonometry is another important branch of geometry in addition to Pythagoras’ theorem that has to be taken into consideration while performing invasive procedures under ultrasound guidance. Sine, cosine and tangent are the basis of trigonometry, they represent the ratio between the sides of a right-angled triangle. Sin(α) is the ratio between side B and the hypotenuse (H) (Fig. 1), Cos(α) is the ratio between side A and the hypotenuse (H), and Tan(α) is the ratio between side B and side A. It is important to note that, for a given angle α, the ratio between the sides is unconnected from the triangle size.

As already stated by Piton [1], in our ultrasound daily practice, we constantly build right-angled triangles: the hypotenuse is the needle route, side A is the distance measured on the skin between needle insertion point and target projection on the skin, and side B is the depth of the target.

Side B is determined by the patient’s anatomy and the operator cannot modify it. As reported by Schulman et al. [2], needle/skin angulation between 30° and 45° is considered to be ideal while performing ultrasound procedure.

Trigonometry is helpful in determining which targets can be reached with such needle angulation. At 45° angulation tan(α) is equal to 1, meaning that side A and side B have to be equal. For example, if our target jugular internal vein is 2 cm deep, the needle has to be inserted 2 cm from the target. Generalizing, we have a good needle visualization whenever Tan(α) ≤ 1 with side B equal to or shorter than side A.

In conclusion, physicians have to take into account target depth while choosing the ultrasound linear probe of appropriate size to be able to visualize all needle routes and the needle skin entrance site.
